# Observations on Man in Health and Disease

**Published:** 1846-07

**Authors:** 


### Etudes de l'Homme dans l'Etat de Santé et dans l'Etat de Maladie. Par *J. H. Reveillé-Parise,* M.D.

### Observations on Man in Health and Disease. By *J. H. Reveillé-Parise,* M.D. 2nd Edition. 2 Vol. pp. 1000. Paris, 1845.

The perusal of any production of the pen of this accomplished physician and able writer always affords us great pleasure. His just appreciation of what should be the noble objects of our profession and the attributes of its members, the importance he attaches to the psychical as distinguished from the mere material investigation and management of disease, the great extent of his professional and classical erudition, the kindness of heart which pervades his pages, and the elegance and ease which mark their composition, combine to render the present two volumes highly attractive, especially to contemplative readers. Several of the papers they contain have before appeared, but are modified by the results of subsequent reflection and experience. "This book," says the author, "is the result of my researches, endeavours, and reflections for more than twenty years, although the fragments which compose it have but very indirect relations with each other, by reason of the variety of the subjects treated. While endeavouring to avoid falling either into scientific common-place or into paradoxical subtlety, I have been desirous of boldly and freely stating my opinion upon many points relating to our art, and then submitting it to the good sense and equity of the public—a tribunal generally just (of course the professional public is here only alluded to by M. P.) and often without appeal. To intolerant and exclusive affirmation, I have preferred calm and reflecting examination, and the search for and legitimate interpretation of facts; being also fully persuaded that it is sometimes advantageous to bring down scientific questions from the loftiness of theory to the reality of application, and sometimes, on the other hand, to mount up by the aid of facts to a rational certitude. Science should not become too materialized, nor should it be allowed to evaporate amid vague principles."

The following are the titles of the various subjects. "The Condition of Health"—"Eclectism in Medicine"—"Management of the Convalescence of Acute Disease"—"On the Imagination as a Cause of Scientific Progress"—"On the Employment of Plates of Lead for the Cicatrization of Wounds"—"On the Science and Profession of Medicine"—"On Moral Medicine"—"On a New Method of Hastening the Cure of Recent Wounds"—"On the Existence and Cause of the Melancholic Temperament"—"Hygiene of the Corset"—"The Basis of the Progress of the Science of Man"—"The Medical Gallery," comprising able sketches of several modern celebrated medical men. Our limited space will prevent our doing justice to all these, besides which, some of the papers hardly admit of compressed notices at all:

### *On the Condition of Health.*

Two opposite errors are usually committed by mankind in reference to their health, the one being the paying insufficient attention to its preservation, and the other the paying too much. By far the greater numbers, however, fall into the first of these errors, and the various fallacies by which they seek to defend their inattention are passed in review by the author; but, as these are capable of refutation at the hands of any professional man, we need not advert to them. " After a long exercise of my profession," says M. R. P. " I am convinced that health is that of which men talk most and care least. *Business before every thing* is, as I have said, their ruling maxim. We are all of us engaged in a cause against Nature, which must be decided before long, and yet scarcely any of us will take the trouble of properly examining the documents upon which so important a decision must rest. Why is this? It is because we eternally forget the grand principle that, it is a hundred or a thousand times more easy to prevent, than to cure diseases."

It may be laid down as a fundamental principle that " *health consists in a normal equilibrium of organic excitement and excitability;*" and, whenever the conditions of the economy exceed or fall short of the limits of such equilibrium, health is menaced. However various organs may be, they all agree in this, the possession of an inherent *excitability,* which would however remain inert and powerless if not developed into action by the agency of another power, usually of external origin, which in its *ensemble* may be termed *excitation* or *excitement.* Although, however, each organ possesses its special stimulus, yet all are united in one consentaneous chain of action, which constitutes that balance between excitement and excitability whence health results. The numerous relations and varying proportions of excitability and excitement, then, constitute the different conditions of the human body, and consequently of its health. In order to better appreciate them, we must not lose sight of the fact that, excitability may be accumulated in any organ if the stimulant be defective. But after the normal relation is once established, if the stimulus is continued or increased, the excitability becomes diminished and exhausted, and the organ, and eventually the general health, becomes impaired. In the first of these cases, excitability being in excess, and excitement defective, we have the *direct* debility of Brown: but where there is diminution and exhaustion of the excitability by the excess and continuance of stimulus, there is *indirect debility*—a more dangerous condition of the economy than the former, since excitement may always be reproduced, while, when excitability is exhausted by age or excess, life becomes extinguished. Among other examples cited is the following. If a man possessing good eye-sight passes from the light into the dark, his eyes fall into a state of extreme excitability or debility: they then become sensible to the slightest ray of light: gradually they become accustomed to this, and support the degree of light suitable to their actual condition. There is a due relation. But let the light be increased in intensity, duration, and activity, and the eye will soon become fatigued: its excitability is exhausted by excess of stimulus, its structure undergoes alteration, and its functions are disturbed or cease, unless such excitement is interrupted.

" In reference to the great influence which this relation exerts on the economy, it may be observed that, however numerous the causes of disease may be in appearance, they may yet be reduced to three principal ones, *wounds, poisons, and moral or physical organic super-excitement.* These causes may act singly or in concert, but the last must be considered as the most dangerous and most frequent. The reason of this is simple and evident. Man avoids the two former as well as he is able; but he frequently advances to meet the other, without reflecting that, in multiplying the intensity of excitement by pleasure or labour, he loses the secret of maintaining the equal proportions of his organic capacity. The allurement of pleasure is especially the rock upon which he splits. Man, great baby as he is, seems continually to be crying out 'give me too much.'

Nature furnishes us with a warning of the violation of the physiological law, whose hygienic consequences we are now considering, viz. in *satiety*. The power which the economy possesses of accommodating itself to different impressions, acts as a remedy held in reserve by Nature for the majority of the accidental evils which external agents can induce. Sometimes this is the natural remedy against pain, of which the perception becomes less vivid according to its duration: sometimes it is a kind of counterpoise (although too often an insufficient one) to that perpetual desire for stimulation which exists in man. However this may be, when excitement has taken place to a certain point, and is repeated, sensation or emotion becomes blunted. What is called the piquancy of novelty and the freshness of impression disappears; but, if the excitement continues, indifference, repulsion, and then disgust and the *surgit amari* of pleasure soon begin to manifest themselves. Now, one of two things takes place: either we desist, and conforming ourselves to the physiological law await the return of excitability, that is we recall the appetite by abstinence—a safe, excellent method, the true economy of life and happiness; or we imprudently continue the excitation. The organism is soon brought into relation with this, and can no longer do without it—without the production of a painful sensation, which is again sought to be relieved by renewed stimulation. This is *habit*, the continued source of unperceived and involuntary acts—a singular, capricious, and incomprehensible condition for those who have not studied the laws of life. Prior to engendering this disposition, man, from an excess of excitability, perceives the necessity of a stimulant, which is *a natural want;* but if he continue this, or augments its quantity or intensity, he is obliged to have recourse to it almost against his will. This is a *factitious want*, the forms of which are infinitely multiplied in our social state. The one disposition dominates over man at least as much as the other; so that the habits form, so to speak, the tissue of our lives woven by us around ourselves. It is a second nature added to the first, and just as tyrannical and as powerful. In fact this second nature, which becomes general in the economy, assumes the condition of an *acquired temperament*, and frequently leaves no power whatever to reason. This factitious, importunate, exacting want is renewed every instant by virtue of the physiological law, that an organ being excited, and become in consequence more excitable, solicits the frequent return of the excitation, and that in an infinite progression. But if the power of a superior will, or external circumstances, do not vanquish this want which has sprung from habit, indirect debility or exhaustion from excess of stimulation, will occur, especially when the grosser instincts of animal life have been resorted to."

The author particularly points out that this excessive excitement may not only result from the indulgence in gross and sensual passions, but also from the inordinate pursuit of objects every way proper in themselves, or indeed of the most elevated character. "The austere pleasures of science themselves are in nowise guaranteed from the effects of excessive excitement, and are just as dangerous as the others, when prudence opposes no boundaries or limits their strength or duration. The passion for knowledge or for art is as devouring, although a far nobler passion than these; and the agitation induced by it produces the same appearance and effects as that arising from love or excessive ambition." The passion which seems to predominate in the greatest excess at our own epoch, "and which seems to gnaw away and waste our existence, enfeebling and exhausting it, is the poignant desire for the rapid acquisition of riches, even at the risk of not being able to enjoy what has been gained or accumulated."

"Never was there seen to the same extent as now the ardent desire to gain, accumulate, and bequeath riches. So is it to be remarked that, certain diseases, as aneurisms of the heart, cerebral congestions, morbid affections of the nervous system, mental alienation, &c. are infinitely more frequent at the present time than formerly, especially in large towns, where they reach a frightful figure. At least in some descriptions of excess prudence struggles, and old age intervenes, and with a man endowed with a little good sense, reason does not entirely loosen hold of the reins, although she sometimes holds them lightly enough. But when ambition, honours, gain, and avarice are in question, the too much is never enough. Age never tempers it; illness scarcely arrests it; and death alone can say 'here are your limits.' But whether we deliver ourselves up to unbridled pleasures, and expose ourselves to the gross slavery of the animal passions, or whether we abandon ourselves no less foolishly to excessive labours of mind and body for the furthering our interests and the acquisition of lucre, it is no less true that, in transgressing the normal type of excitement and excitability, our health becomes compromised in proportion to the degree of excess and constitutional peculiarity."

The following are the applications deducible from the adoption of this equilibrium between excitement and excitability as the law of health. 1. Moderation in every thing. 2. Active exercise of organs. 3. Careful self-study. 4. The special study of preponderating organs. 5. The avoidance of a too uniform life. 6. There should never be several vivid and simultaneous causes of excitement in operation. 7. After vivid excitement the equilibrium should be re-established by intervals of repose proportioned to the intensity and duration of the excitement, and the individual disposition upon which it is exerted. 8. Let habits be acquired or discontinued gradually. 9. The influence of slight causes in the production of important effects on the health must not be lost sight of. 10. Whatever may be the social position of the person hygienic laws must be observed. All these positions are illustrated at considerable length, and with great felicity.

### *On the Characteristics of Eclectism in Medicine.*

"Eclectism is one of the happiest terms which medicine has borrowed from philosophy, because it so perfectly expresses the end in view—*to choose;* and to choose with discernment, after consulting experience, reason and reflection. It is the philosophy of the well-intentioned; of men of a free and pure judgment, who regard every spirit of sect or system as a form of tyranny. The eclectic school is the positive school of our art, the *realism* of medicine. Well then might we be astonished on reading in the *Annales de la Medicine Physiologique,* that eclectism is the opprobrium of medicine. This is the language of a man who has the interests of a system to fight for and defend. Eclectism has indeed always been the cause of dread to the most opposite doctrines, and alike to those systematizers who wish to change the entire face of science, and those who obstruct its progress, or who obstinately deny this. Both agree in repelling this method: they maintain that eclectism is a word void of meaning, and incapable of definition. In spite of this assertion we shall endeavour to propose one.

According to our view, '*Eclectism is the art of estimating the extent and value of proofs;*' and if we mistake not, this definition unites the two characters necessary for a good definition, brevity and clearness. In adopting it we see at once the immense advantages of this method—the sole basis of medical philosophy."

The eclectic method is an impartial one, flattering neither the prejudices or vanities of systematizers. It examines what is true and what is false, rash or unproven in a system. It demands evidence. It is on its guard against the seductions of talent and imagination, and is anxiously occupied in indicating the means of detecting such, by the collection and comparison of facts, independently of the influence of great names and reputations. It admits no doctrine as perfect or exclusively true, but gives its preference according to the extent and value of the proofs that can be adduced. The inventors of systems adopt a different course. They first lay down a general principle, according to which the various facts and consequences are adjusted with more or less success. They all profess to have consulted facts, the very same facts are adduced in defence of the most opposite hypotheses, and twisted and turned to suit the purposes of the preconceived construction. And it is also true that every system may count its cures and its victories; for, first, all is not false in any system; next, we know with what ingenuity reverses are dissimulated or explained; and, lastly, a vast number of patients are so happily organized as to be enabled to resist the effects of whatever treatment they are subjected to. The following parallel may be perused with advantage.

"The systematizer, like the sectarian, rejects every thing which does not proceed from the source he has chosen; there is something bigoted in the plenitude of his conviction. He first asks for the true, then for the probable, and at last admits the absurd. The eclectic endeavours to proceed from the doubtful to the probable and thence to the certain, fortifying himself whenever he can by evidence. Do not ask him under whose colours he serves, to what master he attaches himself, or what is the ensign of his school. A passing visitor, he does not remain wherever he may be carried by a system, but only where reason, experience, and the love of truth conduct him to.

"The systematic having always his master and his doctrine, its progress and its obstacles, before his eyes, struggles in but one direction, and for him the integrity of the dogma is all and all. *Ipse dixit* is the maxim of a slave, says the eclectic, and being neither for Apollos or Cephas, his enthusiasm is excited neither *pro* or *con*. He welcomes the truth from whatever quarter, but he must be sure of its recognition. He must see it, examine every fraction and particle, and convince himself that it is really the truth and not its phantom.

"The fanatical systematic praises or criticizes without reserve, according to the interests of his system; but the eclectic according to the interests of science, and without exceeding a just moderation. With him, pale envy never conceals herself under the mask of the critic. He can recognize important truths in Brown, and delights in attributing to Broussais a rare sagacity in pathological research, and in the art of generalizing facts: but he will not allow that this last author has created medicine as some of his fervent disciples yet love to state.

"The systematic always proceeds from doctrines to facts, while the eclectic does just the contrary, endeavouring to follow the method of the mathematicians, *certum ab incerto, inventum ab inveniendo*. The systematic listens and adopts; the eclectic reasons and deduces: the former engages his obedience and medical faith; the latter never yields his reason to any usurper: the one thinks through an intermedium; the other is always the author of his own judgments. What I believe is the truth, says the systematic. That only is true which has been demonstrated, replies the eclectic. My master declares it is so, say the systematic. What says experience? rejoins his adversary. But, the systematic will exclaim, cannot I see and observe as well as yourself, cannot I apply my faculties to the examination of controverted questions, and conclude in favour of the doctrine I have adopted? No, the eclectic may reply, you neither do or can see as I do; a systematic prejudice perverts your understanding, and it is impossible for you to judge with completeness, coolness, and full knowledge of the cause. A secret inclination, a certain tendency, leads you always to see in facts other than they contain. By the aid of subtelty you can extort from them whatever you please and what even they do not yield. The best disposition for finding the truth is to commence with the destruction of every prejudice, and to become persuaded of one's entire ignorance. Is your judgment pure and disinterested enough to allow of this?

"We may see from this parallel the vast difference that may sometimes exist between two practitioners, supposing them equally well instructed and of equal good faith. And let it not be believed that such a picture is the mere work of fancy; it is the history of the last epoch of science, it was but recently the subject of the most exciting interest in the daily practice of medicine; we read of it every day in our books and in our journals, we have heard it in our schools and our academies. We may also predict which of these two adversaries will eventually triumph. The systematic has on his side the piquancy of novelty, the facility of explanation, and the enthusiasm and number of his adepts. The eclectic relies on time, his most powerful ally, and seldom in vain. The records of science present the most manifest proof that every doctrine, every exclusive system, eventually disappears, in a variable period of time, which upon an average may perhaps be stated at twenty years. We can scarcely except any other than Galenism, and that because it prevailed during the barbarism of the middle ages. The eclectic will be justified by the progress of science; his triumph is founded on the nature of things. He judges but is not judged: because he only clings to that which is demonstrated, and remains in doubt as regards the rest. If he affirms a thing he may be believed, not because he always knows the truth; but because he has taken every pains to assure himself of it, and only affirms what he knows for certain. He may sometimes employ the *perhaps* of the wise man, but at all events he does not seek to impose his own opinions and ideas. And why should he? Does he not know that such opinions must sink or flourish according to their degree of value; and that, as the truth only extends by slow undulations, the circle, enlarging more and more, will in the end excite the attention of enlightened men? Time and perseverance only are wanted."

The systematics declare, that however well-looking eclectism may be upon paper it is destitute of practical applications. The author replies to this by exhibiting several instances in which such have been advantageously made. Thus, although they allow that in many cases essential fevers are but phlegmasiæ with resulting general irritation, the eclectics maintain that an intermittent fever is something very different from this. They admit the localization of many fevers but reject the Broussaian doctrine of gastro-enterite as a general explanation. They have indicated also that chronic gastritis, which recently created so much attention, has often been mistaken for gastralgia. In reference to tubercles, again, eclectism is as impartial, for while denying their origin in the irritation of the pulmonary lymphatic ganglions, it admits that inflammation performs a certain part, as yet not to be appreciated, in their development, progress, &c. Eclectism acknowledges the importance of the organic lesions observed after death, but it will not allow that pathological anatomy should be made the sole basis of medicine—to the neglect of the primary changes which the fluids undergo. Eclectism exacts, besides a spirit free from all servitude, great power of discernment, for the seizure of the truth when it offers itself to view; great knowledge, for the employment of terms of comparison; patient research, in order to examine and probe everything to the bottom; and untiring, inexhaustible, attention, because nothing must be rejected without a thorough examination. Such acquisitions are rarely found united in one person; but the habit of seeing patients, the exertion of good sense, and a kind of intuitive evidence, do much towards forming the eclectic practitioner: while the accumulated experience of many such, forms a powerful instrument for the overthrow of any exclusive system, even while retaining the truths it may have contained and perfected. Academies and learned societies are in the essence and object of their institution Eclectics.

Eclectism, it is said, leads to scepticism, but surely doubt in so uncertain a science as medicine is no fault. It has been declared also, that it is too exacting, neglecting important facts because but little known. In truth, however, it neglects no fact, however slight, that can be proved to be one, but declines receiving hypothesis and conjecture in lieu of demonstration. Of what use is eclectism? say others, it invents nothing! This is true. The eclectic does not plant and sow, but reaps and sifts. And is not the proof of the veracity of a fact as useful as the discovery of a new one? The eclectic confers another advantage by becoming the mediator between the most opposite sects. He is suspicious of all extreme opinions alike, but opposes none with bitter hostility. He is equally averse to the obstinate adherence to exploded doctrines and practices, and to the ardent unreflecting adoption of new ones. Experience is his only law and medical creed. The virulent opposition of the advocates of the various systems is the less justifiable, since eclectism is often the only means of preserving the truths they have really acquired, and maintaining their reputation for the progress they have facilitated. From the impartiality of eclectism alone can such justice be obtained. The eclectics, without being alarmed at the overbearing language of Broussais, have always done justice to his merits, although they have deemed it necessary to submit his various doctrines to the test of experience, and not to receive them upon his mere affirmation.

We have transcribed our author's statements at some length, not only because they are in themselves interesting, but because they point to the characteristics that so pre-eminently distinguish modern English practice, which has always shewn itself averse to succumb to the requirements of any exclusive system. In advocating eclectism, however, M. Reveillé-Parise by no means undervalues the legitimate uses of theory and hypothesis, but accords to them a very high value. He says:

"The anathemas launched on all occasions against systems, appear to us but as mere declamation. Our object should be to appreciate, not proscribe them. There must be systems in medicine, for nothing will ever be discovered but by endeavouring and by *imagining;* for to leave the field of science at rest is to condemn it to sterility. Little does it signify to us that sectarians declare their doctrine as the only true one, and consequently the only durable one, for this is the common sophism which only deceives him who desires to be deceived. In fact, who is ignorant that a system in medicine is a tissue of errors and truths more or less logically disposed, all seeming united by a general principle, that favorable and approving facts are alike collected, forcibly arranged within the frame-work, and attached to the theory, and that their interpretation is usually arbitrary, often contradictory? What matters this? If the evil is great it is temporary, while the good will remain."

In a subsequent essay, consisting of two letters "*Upon the Imagination as a Cause of Scientific Progress*," the author pursues this argument at great length and with varied illustration. He shews that, without the power of imagination and invention, no great discovery or progress in science ever has, or ever will be, made: and that all great improvers of science, as Bacon, Newton, Linnæus, Harvey, Davy, &c. have been pre-eminent in this respect. Our space prevents our following him through his demonstration, but we will extract one passage.

"I am not here speaking of blowing mere soap-bubbles termed hypotheses, but of theories with sufficient basis, the true expression, as near as possible, of observed phenomena. Nevertheless, we must not be frightened at every attempt. For my own part I plainly state again that, without hypothesis, the terror of little minds, science would be eternally confined to a narrow empiricism; if systems were not invented, which, without being the truth themselves, are a preparation or preliminary to it; and if we must only see, smell, feel, measure, and rely upon our senses, we deliver ourselves up to insufficient guides, stop at the semblance, and desert the philosophy of science. And yet the highest abstractions of the science of man are not to be neglected. In fact, to idealize or abstract, is to comprehend the reality better than vulgar minds, for it is to seize the hidden connection of things and facts, of causes and manifestations. Nevertheless, this word *imagination* inspires distrust, because its value in science is not known. It is believed that it is concerned only with absurd visions; but there cannot be a greater error. I allow that nothing can be more injurious to science than systematising at first view, and generalizing by supposition; but not generalizing at all, and remaining content with material facts, has also its peril. I have not denied that utopias, chimeras, and dreams belong to the family of systems, but so also do enlarged views, principles, fundamental axioms, action, movement, and progress. Imagination sometimes wanders too far in the field of conjecture, but with experimentalism alone, its self-infatuation, its confined logic, more or less supported by statistics and sophisms, truth is not discovered but disguised, and is lost in uncertain deductions, contradictory assertions, and columns of figures."

### *On the Management of the Convalescence of Acute Disease.*

M. Parise observes that most medical histories terminate with the entrance of the patient upon the convalescent stage; but convalescence is a very different state to that of health. After a serious and prolonged illness every organ suffers more or less from exhaustion, and their functions are feebly and inharmoniously performed. This condition of the economy results from the violent excitement produced by the disease, whether this has been febrile or not, and the privation of food. The former of course no longer operates, and food may now be administered for the purpose of replenishing the supply of blood, and hence recruiting the forces of the economy. The *stomach* is therefore the organ with which we have to do.

When an organ has been long deprived of its accustomed stimulus, *its tone becomes diminished and its sensibility increased,* supposing no organic lesion be present; and so it is with regard to the stomach of convalescents, and that in proportion as the disease has been prolonged and grave.

Much more so is this the case when the disease itself has been principally located. in the digestive organs. We may lay it down, then, as a fixed principle, that in every convalescence *the sensibility of the stomach and intestines is augmented, and their contractility diminished.* The indications are therefore obvious enough. Diminish the sensibility of the stomach, augment its tone, and consequently enable digestion to be properly performed: but our means of accomplishing these are not so efficacious as might be supposed. If the subject be young and robust, with vigorous digestive organs, health is soon restored, and digestion established; but even in such a one, if the tormenting hunger which accompanies convalescence be sought to be appeased by injudicious supplies of food, various disturbances of the digestive organs ensue. "But if the convalescent is naturally delicate, nervous, or irritable, as often happens in the case of literary persons, artists, &c., if his digestive powers are none of the strongest even in health, if he has reached a certain age, or has been worn by anxiety and sorrow, we must expect a tedious and difficult convalescence. You believe you have gained your object, and suddenly find yourself far removed from it. During this period, the economy of the invalid suffers and languishes, his impoverished blood is slightly or badly replenished, and he continues pale, serous, and devoid of plasticity. The more this condition is prolonged, the more pronounced is the gastric sensibility, as also even his individual sensibility; for the old axiom of practical medicine, 'the blood is the regulator of the nerves,' is verified by every day's clinical experience."

At first sight, the mere *fortifying* the stomach by the administration of tonics would seem to be the simple means of procedure: but every reflecting practitioner has been in the condition to observe numerous cases in which the irritable and sensitive condition of the stomach renders the establishment of its digestive power a matter of nicety and difficulty. If we employ soothing or calming means and a debilitating regimen, the tonic power of the organ is gradually diminished; while, if we have recourse to stimulants, uneasiness, gastric irritation, thirst, dryness of the mouth, &c., prove we are exciting the organ to excess. *Diarrhœa* occurring during convalescence oftentimes deceives much. For we may have difficulty in determining whether it depends upon the remains of irritation of the digestive tube, augmented by injudicious diet, or upon simple atony of the canal. Tact is oftentimes the practitioner's sole guide; but, as a general rule it may be stated, that the diarrhœa of convalescence is connected with a defective tonicity, which will be yet further diminished by the abstinence and local depletion too indiscriminately resorted to by some practitioners.

Our means for establishing the digestive powers must in general be neither of a debilitating or of a too stimulating character; but adapted, in their nature and quality, as near as possible to the degree of sensibility of the organs. The following indications may then form the basis of the convalescent regimen.

1. *Allow only as much food as the stomach can digest.*—Above all, we must not proportion the amount of this to that of the hunger of the convalescent. The *appetite* here continually provokes the admission of more food than the stomach can dispose of. The patient ought not to experience weight at the stomach, flatulence, but a sensation of comfort, during digestion. The defæcation should be especially attended to. If the matters are excreted in small but well-formed masses, or if there is even slight constipation, the convalescence proceeds favourably. But when all these favourable circumstances are not present, we have to fear the manifestation of serious accidents. "We frequently observed this in the tedious and difficult convalescence of cholera morbus. Supposing even there be no relapse, a repetition of laborious digestions impresses its peculiar mark of weakness and uneasiness. *Cacochylia*, (the old and very just term for an ill-elaborated chyle,) replenishes neither the blood, the strength, or the organic energy."

2. *The patient should eat little and often.*—Trite as this rule is, it is one of the highest importance. A feeble and irritable stomach can neither endure entire abstinence or repletion. So that, while you do not gratify a ravenous appetite with a too-large quantity of food, if you condemn the convalescent to the torments of hunger, you engender such a susceptibility of the stomach that even a very moderate quantity of food cannot be digested. Immediately that the stomach vigorously seeks for aliment you must furnish it with this, even in the night, but only in small quantity. The exact number of times food is to be given, must of course depend upon the conditions of the stomach, age, stage of convalescence, &c.

3. *Submit the food to effectual mastication.*—The importance of this rule for securing easy digestion is generally acknowledged—"He who chews little and swallows rapidly, should have a stomach of iron. Frequently, however, the convalescent, urged on by hunger, allows scarcely any time for mastication; whence result indigestion, diarrhœa, and an interminable convalescence."

4. *Let the patient be maintained warm, and especially in his feet, during digestion.*—Delicate persons frequently experience a rigor after meals, and this is especially the case with the convalescent, in whom the sensibility is great and the quantity of blood small. Cold feet are especially dangerous for delicate and irritable persons, and the convalescence may continue long after severe diseases before the animal heat is generated with sufficient energy to penetrate to the extremities of the body. Until then, it must be artificially assisted by friction, warm yet light clothing, double shoes, &c.

5. *Let the nature of the food be adapted to the sympathies of the stomach.*—No organ is so capricious in its likings and aversions as the stomach; and the diet, therefore, requires adaptation to individval peculiarities, the habits and tastes of the patient being always consulted when these are not essentially hurtful. The choice of food from its containing the greatest amount of nutritive matter in the smallest space cannot be established as a rule of universal propriety. Some persons cannot bear fatty, saccharine, or herbaceous articles of diet, while these suit others exactly. Some demand light aliments, and others prefer those of a substantial, or even heavy character, giving a sense of fulness and support to the stomach.

“ In 1810 the regiment to which I was attached in Spain arrived early one morning at a little village in Arragon. As there was neither time nor means for the preparation of the coarse ammunition bread, a requisition was made upon the inhabitants, and double rations of a very beautiful white bread was distributed. In the evening, however, the soldiers declared they were dying with hunger. ‘How is this,’ said the colonel to a grenadier, ‘did you not receive a double ration this morning?’ ‘That is true enough, colonel,’ replied the old soldier, ‘but this *muslin bread* has passed through us so quickly, that we do not know what has become of it.’ ”

6. *Variation of the diet.*—Much contributes to the facility with which it is digested; but it must be managed with prudence, for woe to the convalescent, who, guided only by his gluttony, delivers himself up not only to his appetite, but even to the caprices of his appetite. All that flatters taste and sensation is not suitable for the stomach.

6. *Change of air.*—Sometimes in spite of every measure, however, methodically put into force, the convalescent gains no ground. The digestive canal remains inert and feeble, without any cause manifesting itself. In such cases, change of air is of marked efficacy, “ even where the atmosphere in which the patient resides possesses every condition of salubrity. I have seen ameliorations produced by the patient but changing his abode from one part of Paris to another.” Of course country air is far preferable in the great majority of cases.

7. *Let vivid moral emotions be avoided.*—The injurious effect which sudden moral emotions, whether pleasurable, or the contrary, exert upon the digestive powers is well known to the practitioner.

“ This effect is far more marked in the state of convalescence, when morbid nervous susceptibility being in its highest condition of exaltation, demands long-continued and well-directed precautions. There is not a practitioner in Paris, who, during the epidemic of Cholera in 1832, had not occasion to observe convalescence impeded by moral causes. Observe, also, that these causes were sometimes slight, and bore no relation to the accidents they gave rise to. I have seen mortal relapses induced by the breaking of a porcelain vase, an annoying noise, the falling of an insignificant letter into the fire, &c. Moreover, we may remark that, in some subjects these moral causes operate secretly. The patients are enabled to smother their grief, and restrain their tears, but the pathological influence is no less certain and dangerous. It is for the physician to discover the secret causes of this precordial anguish, to remove them, or at all events to endeavour to diminish their effects. But to attain this end he will have to combine with his knowledge, an exquisite tact and a practical acquaintance with the human heart. Hold out hope! incessant hope, be prodigal in this! it is the veritable charm for disease, *incantatio malorum.*”

Whatever skill and care may be employed, the progress of the patient may still be impeded by the occurrence of various accidents. Only two are alluded to by M. Parise, and that because of their frequency, viz. *diarrhœa* and *gastro-enteralgia.* When the first of these is present, we must endeavour to discover whether it is produced by errors of regimen, a chill, or by some moral cause. Is there any inflammation or disorganization, or simple atony of fibre? Even where from the symptoms we believe inflammatory action to be present, we must still be very cautious in recommending complete abstinence and leeching, which may produce a degree of exhaustion or contractility of the parts, that may require years for its reparation. Diminution of food will, however, then be required, as well as mild counter-irritation applied in the form of dry-cupping, sinapisms, and frictions to the abdomen. When the irritation has subsided, mild tonics and a gradually improved diet are indicated. When the diarrhœa is *passive*, tonics combined with antispasmodics may be given in very decided doses. M. R. P. speaks highly of the utility of *theriacum*, and especially when united with calumba. *Diascordium* is often useful, but sometimes too stimulating. Aluminized and laudanized waters, or small doses of acetate of lead, also cure the affection. The following formula is recommended by the author. ℞. *Mucous Extract of Opium*, gr. j. *Powder Acacia*, 12 grs.; *Powder of Calumba*, ℈ij.; *Mint Sugar*, ℈iv. *mix for six doses*. The employment of light wine diluted with sweet infusion of lime or orange leaves or camomile; the drinking but small quantities of fluid; the selecting aliments as dry as possible, as crusty or toasted bread in preference to crumb; and the taking food instantly craving is felt, are among the practices recommended.

*Gastralgia or enteralgia* is of far more frequent occurrence after severe disease, especially when the alimentary canal has been implicated, than is generally believed. The irregularity of its attacks forms one of its most distinguishing features. The poignant feeling of hunger, sometimes so acute in this disease, suddenly changes into a state of insupportable "gastric languor." In this disease, a superabundant nourishment is injurious, but in a far less degree than a too scanty one. Abstinence cannot be borne, nor do the epigastric pain and traction cease until a certain quantum of food has been swallowed; while, if its administration be too long delayed, digestion does not take place, and diarrhœa is produced. "Woe be to the patient whose attendant mistakes a gastralgia for a gastritis!" First among medicines adapted for gastralgia may be named the *sub-nitrate of bismuth* (indeed an admirable remedy in this class of affections) given alone or with opium or calumba. External revulsives, as blisters, cupping-glasses, or sinapisms, should be applied to the abdomen. The endermic use of morphia is often very useful; as are also equitation, gymnastics, travelling, &c.

Whatever means we have recourse to for the restoration of the energy of the digestive process and the establishment of the normal equilibrium between the sensibility and contractility of the canal, a prolonged employment of a variety of measures is demanded. "Perseverance and variety must never be lost sight of by the sagacious and prudent physician in an obstinate, troublesome, convalescence, during which health and disease are constantly vibrating."

### *On the Employment of Plates of Lead in the dressing of Wounds and Ulcers about to cicatrize.*

No one has oftener than the military surgeon occasion to exemplify the truth of the proverb, that necessity is the mother of invention; and our author, finding himself destitute of numerous appliances at the siege of

Saragossa, 1808-9, was obliged to tax his ingenuity to discover appropriate substitutes. The want of *lint*, however, was his most serious privation, for although moss and heath served to fill the pads for compound fractures, and tow for the dressing of large and abundantly suppurating wounds, yet there was no means of supplying the place of fine lint in dressing granulating, sensitive, sores. He was, in fact, obliged to leave such uncovered, until the happy idea struck him that thin leaden plates might serve as a protection for the surface. Balls there were in abundance, and the author and several soldiers set to work with all their energy to hammer these out into plates, some of which were no thicker than a leaf of paper. To his surprise and satisfaction he found that these not only did not oppose but favoured cicatrization; and since that period he has continued to derive the greatest advantage from their employment.

A wound which is disposed to heal requires only that its surface should be protected while Nature conducts the healing process. LINT is an excellent protection of this kind, maintaining also a mild temperature of the part favourable to its union. It is also an excellent means of absorbing the pus, when this is secreted in too large a quantity. The moderately stimulating effect which some suppose it to exert upon the wound, thereby hastening its healing, is very doubtful. Among the inconveniences of this substance is the difficulty of obtaining it of sufficient good quality, at least when large quantities, as in armies and hospitals, are required. If it is too coarse and hard it irritates and inflames the wounds, while, when too fine and thin, it adheres to them so tenaciously, as to be removed even by soaking only with difficulty, often detaching the tender cuticle with it. The edges of the wounds are kept bleeding and irritated, and cicatrization is retarded. The lint sent to the army, from bad packing or selection, often proves a source of great irritation to wounds, while it has not unfrequently, when washed again to be used, been the means of maintaining hospital gangrene and other epidemic affections. The increased and increasing price of this article renders the discovery of a cheaper substitute also very desirable, when very large quantities are in request. The adherence of lint to the edges of wounds has been sought to be prevented by the intervention of strips of cerate. But the thin cicatrizing edge of a wound is eminently sensitive, and the re-iterated application of greasy bodies to it often induces pruritus, or almost even erysipelas, especially when the ointment is at all rancid. Moreover, the removal of the layers of these fatty bodies which cake on the edges is often very irritating. Prepared tow has of late been much substituted for lint, but it very imperfectly supplies its place.

The employment of leaden plates seems much more fitting for favouring the cicatrization than any other means. They are to be retained *in situ* by compresses and bandages, or by adhesive plaister. Their suppleness and flexibility, and the ease with which they may be cut or shaped into any form, render them a very convenient and expeditious mode of dressing wounds. The action of the lead is purely mechanical, for the same effects may be obtained by plates of tin, gold, or silver, but its ductility and cheapness render it the most eligible substance. As the lead contracts no adherence with the edges of the wound, fatty bodies are not required, the dressing is far less painful, and the newly-formed skin is in nowise irritated or torn. The dressings are also required to be renewed only every two or three days, according to the quantity of the discharge and irritability of the wound. As Nature is thus secured from interruption, the work of cicatrization proceeds much more rapidly. Infection, moreover, can never be communicated, as it may be by the employment of old lint; for the same plate, if not too thin, may be used for successive patients with perfect safety, if the precaution be taken to have it well cleaned and slightly polished. Many practitioners have tried this plan of treating wounds since it was first proposed by the author, and have confirmed his statements of its efficacy. Some, however, believing he had recommended it only for ulcers, have failed in deriving advantage from it. His object was, however, different.

"From the title I have given to this essay it is easy to see that I allude to wounds and ulcers only which are disposed to cicatrize, that is to large, superficial, red, granulating, painless wounds, covered with a fibro-purulent layer, and surrounded by a deep, rose-red margin. On the contrary, when the wound is deep, when it does not present any of the conditions favourable to a speedy cicatrization, if there is pain, whencesoever this may arise, if there is abundant suppuration, or if the wound is kept open by a virus, and it is deemed desirable to treat it by local applications, such as cataplasms, &c., I do not think this mode of dressing is ever attended with success, at least such is the result of the numerous trials which I have made. Nevertheless, it is proper to have recourse to it whenever, the obstacles to cicatrization being removed, the solution of continuity tends to a cure. In spite of these numerous exceptions, this mode of dressing will thus prove suitable to the great majority of wounds; and farther than this, there are cases in which it has succeeded, although in a different manner to that already alluded to. Thus, in certain examples of abscesses forming in encysted tumours, after the contents have been evacuated through a small aperture by the aid of the cupping-pump, a strong leaden plate bound over the part, has in a short time effected a cure, by producing adhesion of the walls of the abscess. To return to our proper subject. Whenever a wound is brought into a simple condition, and the obstacles to its cicatrization no longer exist, this is the best of all dressing, and its success is certain. However, *large and superficial wounds* are those in which it has seemed to me to be most suitable. *Burns* offer a remarkable example of this. If they are slight we may at once resort to the lead; but if they are deep we must wait until the consequent suppuration has sufficiently diminished. In both cases, the leaden plates, applied at more or less long intervals, will effect a cure of the wounds, as they will also those resulting from the application of *blisters* in irritable subjects, or at the end of long diseases. Many practitioners have observed that patients in these cases bear the application of lint or cerate to the ulcerated surface with great difficulty. Every dressing is accompanied by adhesions, bleeding, excessive suffering, loss of rest, fever, and delay of cicatrization. A simple leaden plate, which is occasionally removed, at once remedies all inconveniences of this kind.

"This mode of dressing succeeds equally well in wounds situated upon certain parts where cicatrization is usually obtained with difficulty, as over the elbow, the shin-bone, the ankle, and the tendo-Achilles. Every surgeon must have experienced the difficulty and anxiety which sometimes attend the endeavour to produce cicatrization of even small wounds situated on these parts. This new plan effects this with certainty. * * * * There are wounds *with loss of muscular substance* which no means will cicatrize, and the leaden plates offer no particular advantage in this respect: but the dressing by their aid is far more promptly and conveniently performed than with lint, while they offer a kind of artificial integument as a substitute for the natural one.

"No one is unaware that *large cicatrices* are very easily torn, and that the healing of the part is subsequently obtained only with great difficulty. In these cases the dressing with lint and ointments irritates the newly-formed skin, and re-opens the wound again and again, so that this is at last sometimes deemed incurable. But under the use of the leaden plate the healing is completed, although requiring a longer period than under other circumstances. Moreover, a new cicatrix may be rendered more firm, and is seldom then torn, by protecting it with one of these plates."

The wounds, ulcers, or erosions which form upon *œdematous extremities* are also advantageously managed in the same mode. So also the spreading ulcers which follow the application of *blisters in children.* Sometimes, after an *issue* has been made, it cannot be kept open by reason of an erysipelatous redness which surrounds it, and which is usually increased by ointments, &c. The application of a leaden leaf in a short time allays all irritation. As regards *ulcers,* properly so called, the lead will not accelerate cicatrization if they are kept open by any special local or general cause which first requires removal. In *varicose ulcers,* however, combined with pressure, the leaden plate is highly useful. To secure prompt success in the use of the lead, the plates must be properly selected, so that, without being too thick, they will not become torn upon the least movement. The tin-foil which surrounds chocolate, has been endeavoured to be used as a substitute ; but even when trebled or quadrupled it offers scarcely consistence enough for repeated use. The plate must of course be accurately adjusted to the wound, just as any other dressing requires to be. Some surgeons have advantageously employed the leaden plates in even abundantly *suppurating wounds,* piercing them with a large pin in many places to give exit to the matter.

As connected with the foregoing subject, we may here notice the author's other paper upon the treatment of wounds. It is entitled—

*A new Method of expediting the Healing of recent Wounds.*

As the method has already been published in one of the French journals and copied into our own, we need here only recall the principal points set forth. The great object in the management of wounds is the *prevention or diminution of inflammation.* Wounds only of a very slight extent and depth, unaccompanied by complication, have hitherto been considered as capable of union by the first intention. This arises from the presence of a foreign body, which, whether solid or fluid, must be eliminated by the suppurative process before union can take place. Foreign bodies, however, impeding union, do not always proceed from without, nor are they always perceptible to the senses, and capable of removal by instruments, &c. Effused or infiltrated blood, the little clots closing up the divided mouths of small vessels, fragments of skin, cellular tissue, or torn and bruised muscle, may produce all the effects of the ordinary foreign bodies. A *detritus* of this kind induces irritation, inflammation, and suppuration ; and what the older surgeons termed the *detersion* of a wound is only an expression for the elaboration of these foreign bodies by the efforts of Nature, and the consequent placing the wound in a condition for healing. In proportion as the wound is made by a sharp instrument, and is of a clean uncontused character, the amount of *detritus* will be smaller, and the chances of adhesive inflammation greater. Thus fire-arms, by the great amount of contusion they induce, produce the description of wound least favourable for prompt union.

" In scientific investigations we should never lose sight of the principle of proceeding from what is real and proved by experience to possible amelioration. Experience here proves that the more considerable is the *detritus* of a wound, the less hopes we can entertain of a cure without inflammation or suppuration. The primary indication is then, at first, either to seek a cure by the first intention, or at least to diminish, as much as possible, the inflammatory action which is to follow. The approaching the edges of the wound, the removal of atmospheric influences, the extraction of external foreign bodies, the support of any flaps that may exist, occasionally the use of compression, and, especially in recent times, the more or less prolonged employment of refrigerants, are the means daily resorted to with more or less success. For my part, I believe that, before having recourse to measures for diminishing inflammatory action, and especially the employment of cold water, there is a more fundamental indication which requires great consideration, viz. the *immediate and complete removal of the detritus.* Nothing is so likely to prevent or diminish inflammation as the consequent suppuration. But how is it to be acomplished? The best means is by the exercising a strong aspiration upon the surface of the wound, whether this be done by means of *suction* as employed by the ancients, or by the application of the *cupping-pump* as I have practised it myself. By this procedure the effused blood is *pressed out* from the wound as well as from the extremity of the small vessels, and from the cellular tissue. The same occurs also with regard to a number of small, almost invisible, fragments, furnished by the tearing of the parts. When no foreign body any longer exists, and the wound remains clean and pure, if we can so speak, no cause of irritation being present, there is no inflammation, or, if this cannot be entirely prevented, it is at least much limited in its intensity and consequences—of course taking into account the obstacles to union which the gravity of the wound and the nature of the organs affected may offer."

The suction of wounds was employed in the most ancient times, and even a class of persons devoted to its performance existed. Their offices were chiefly required for the bites of serpents, but were also sometimes employed after the wounds of arrows, spears, &c.; and Percy states that, in the last century, there were men in the French army whose duty it was to perform this suction. At Amsterdam, a treatise on the " Art of Sucking Wounds" was published in 1712. There can be no doubt that suction and cupping-glasses have been advantageously used in the management of poisoned wounds ; and the observations of Celsus in favour of the practice have been amply confirmed by modern observers. In simple wounds, also, the author has convinced himself, by experiments on animals, that the application of the cupping-glass much hastens the curative process.

In employing this means the cup used should always be rather larger than the surface of the wound, so that this latter be completely submitted to the action of the pump. The application, too, should be resorted to as quickly as possible, for if enough time have elapsed to allow of the setting up of irritation or inflammation, the operation is contra-indicated. The pain occasioned is very slight and bearable, and any bleeding which may be produced ceases after the glass is removed. It is almost needless to add, that this means does not exclude the employment of any of those ordinarily had recourse to. M. Parise cites several instances, in which the healing of wounds of various degrees of gravity, as regards the accompanying contusion of parts, seemed to have been much accelerated by the employment of this means. He suggests the following extension of the application.

"I have not had the opportunity of employing this means after serious and important surgical operations, but I am disposed to believe in its utility in diminishing the intensity of the inflammation, and hastening cicatrization in these cases. Probability, which ordinarily foresees more than it sees, here is increased almost to a certainty, for the same causes produce the same effects. I am indeed convinced, that if, after the amputation of a large limb, the vessels being tied or submitted to torsion, a large cupping-pump were applied to the surface of the wound to remove the *detritus,* prior to dressing the stump, the subsequent inflammation and suppuration would be much diminished. Perhaps, too, purulent infection, that deplorable accident which carries off so many patients, might be thus prevented. Where would there be the danger in the experiment?"

### *On the Melancholic Temperament.*

Notwithstanding the revolutions which medicine has undergone, the temperaments have remained much as the ancients described them, and so faithful to nature have the description of these been, that the efforts of Stahl, Cabanis, Hallé, and other celebrated modern physicians, have only succeeded in impressing certain modifications upon these. "The ancients founded their doctrines upon the condition of the fluids—the moderns have considered only the arteries, veins, and lymphatics; the one founding their doctrine upon the contents, and the other upon the containing parts: they were humorists, and we are solidists: and this is all the difference. We always must refer to their groups of the characteristics of temperaments, modified however by the progress of science." The *melancholic temperament* has been however among the moderns a subject of much controversy. The doctrine of Galen which prevailed in the schools for 16 centuries explained it by the existence of *black bile,* without, however, explaining what was to be understood by this. Cabanis and others look upon it as only a variety of the bilious temperament, without shewing in what it differs from this. Tissot and some others denied its existence altogether, while Broussais referred it to a chronic inflammation of the digestive organs.

The author believes the characters of this temperament to be well-marked, and that the denial of its existence became its cause has not been ascertained, is a very unphilosophical procedure. He has frequently met with it in Italy and Spain, and it is not uncommon in France and England; but, as a general rule, it most frequently occurs in hot and dry countries, as India, Spain, Arabia, &c.

"Political and religious institutions may develop or exaggerate it, but they cannot produce it; for we must not confound *melancholy* with the *melancholic temperament*; the former may occur in every variety of organization, while the latter only predisposes to this singular neuralgia.

"Slender forms; muscles slightly developed, their fibres being however dry and rigid; a narrow chest; a yellowish-brown skin covered with hairs, and generally brown ones; large veins coursing over the cutaneous surface; compact cellular tissue; a thin, bony, pale, or olive-coloured face; eyes sometimes blue, but usually black and deep-seated, having the conjunctiva of a yellowish colour; a timid, but sometimes sparkling look; slow, circumspect, and embarrassed movements—are the external characteristics. The internal ones are marked by a quiet, small, contracted, and sometimes unequal pulse; feeble respiration, inactive hæmatosis, and scanty secretions, especially that of the skin. The digestion is very uncertain, while some epigastric tenderness is often present.

"The cerebral system and intellectual faculties correspond to those conditions of the organism. The will is at first uncertain, then obstinate and inflexible; there is an extraordinary perseverance in habits, and a distrust which only increases by acquaintance with mankind: sometimes there is great feebleness of intellect, and at others great power and depth of thought and observation. The imagination always runs to extremes, and, capable of producing chimeras and chefs-d'œuvre, it takes on a double aspect peculiar to this temperament. It is at once vivid and capricious, and yet able to strongly concentrate itself in objects submitted to meditation, so as to be able to follow a fixed idea to the extreme limits of reflection."

As the *sanguine* temperament is the result of the predominance of the *arterial* or red-blood system, so is the *melancholic* produced by the predominance of the *venous* or *black-blood* system. Upon opening the body of a man of this last temperament we are struck with the brownish-yellow colour of his tissues, and the slight development of the various organs. *Venous plethora* is observed in the chest, but particularly in the abdomen, the portal system being especially loaded. The blood contained in these engorged vessels is also remarkably black. The physiological characters already given as those of this temperament, are readily explained by this predominance of the venous system, *e. g.* the colour and difficult transpiration of the skin, the defective hæmatosis, small and slow pulse, abdominal uneasiness, &c. The predominance of the sanguine and lymphatic temperaments in *women* explain also the rarity with which the melancholic temperament is observed in them: so, too, in reference to *age*, none of the temperaments depend so much upon the influence of this in exhibiting their characters; and where these are witnessed early, so are other signs of old age prematurely observed. Fernel fixes the 65th year as the period when the melancholic temperament is observed. Cullen believes that venous plethora commences between the 35th and 40th years. The period when it manifests itself doubtless differs according to original constitution. Its advent is announced, besides the external signs already adverted to, by two anatomical changes consequent on the progress of age, viz. the diminution of the capacity of the chest with ossification of its cartilages, and the diminution in the size, elasticity, and suppleness of the arterial system, the veins becoming at the same time enlarged and distended. All men, therefore, in the progress of age, acquire this temperament, but in very different degrees.

The melancholic must not be confounded with the *bilious* temperament.

"In the latter, the chest is broad and ample, giving rise to great development of the lungs, whence an active hæmatosis, a voluminous, active heart, and an ample arterial system. The muscles are thick and well defined. The secretion of fat increases abundantly with age in the bilious but not in the melancholic. The *hepatico-gastric* system always plays a large part in the former, while in the latter, there is rather an abdominal venous ingurgitation. In the one, there is bilious vomiting and the class of diseases attributed by Stohl to *polycholia,* while, in the other, hæmatemesis, melæna, hæmorrhoids, and passive hæmorrhages in general, are far more frequent.

"The moral contrast is as strong. The man of unmixed bilious temperament is prompt, active, decided, fiery and irascible, he can conceal nothing: resistance is odious to him, and contradiction insupportable. He attacks obstacles with directness and hardihood. His language and manners announce the impetuosity and violence of his organic movements."

It is equally distinct from the *lymphatic.*

"The physiologico-anatomical considerations we have stated shew that the melancholic temp. is never allied with the lymphatic. The vascular system containing the white fluids is only very greatly developed in early life, its activity diminishing with age. It is completely and in everything the opposite of the venous system. We see *lymphatico-sanguine* and *bilio-sanguine* temp., but the characters of the melancholic and of the sanguine are never confounded. They are mutually exclusive; only one of the two great arterial and venous trees can predominate in the economy."

M. Parise enters at some length into the modifications produced upon this melancholic or venous temperament by different degrees of development of the *nervous system;* and then proceeds to consider some of the corroborations of his views derived from pathology and clinical experience. The influence which the venous predominance exerts upon the vital actions must be, in some degree, extended to morbid affections; and thus we find in the melancholic temperament acute affections are less common and less severe, and chronic ones more common and more obstinate than in other constitutions. Inflammations are often latent, and may be attended by disorganizing processes not discoverable until after death. *Melancholia,* which is dependent on a certain condition of the nervous system, may occur in any temperament, but it is the melancholic which especially predisposes to it. The frequent connexion of this affection with the melancholic temperament is shewn by the marked relief which frequently follows the disgorging the abdominal venous system by hæmorrhoidal flux or other discharges, either natural, or artificially induced by leeches or purgatives.

"It is a remark made long since by practitioners, that each age has its hæmorrhages. In infancy and childhood they are manifested in the upper parts of the body, in manhood and old age in the lower parts. We see now how easily this is accounted for. We see also why hæmorrhoids are so salutary to a man predisposed to apoplexy. Whenever, in such a constitution, there is no varicose dilatation of the hæmorrhoidal vessels, the hæmorrhagic effort is concentrated upon the encephalon, instead of being directed towards the abdomen and lower portions of the body; hence apoplexy, partial paralysis, &c. Let us not forget that the impulsive force, acting upon the venous blood, is almost of no amount, and diminishes progressively. This is particularly the case in the brain, notwithstanding Nature's contrivances for facilitating the circulation of the venous blood by multiplying the sinuses; and thus cerebral sanguineous congestions are very frequent, especially in old age, whether on account of the abundance of black blood, or the weakness of the very extensible and dilatable venous tissue. Veins have a general tendency to become varicose, owing to the slight elasticity of their walls. When this occurs to the superficial veins, we apply appropriate measures, especially compression; but when such dilatations occur in the viscera, and especially the brain, where venous plethora may so easily arise, is so common and so dangerous in mature age, it is impossible but serious consequences must result. In this way we explain the occurrence of apoplexy in old men, and why thin, non-sanguineous looking, persons sometimes are liable to cerebral congestions."

The same condition of the venous system influences the diseases of the nervous system, and may give rise to hypochondriacism, monomania, &c. The venous temperament likewise predisposes to *gangrena senilis*, and the passive dropsies so common in advanced age.

The author concludes his paper by a few observations upon the utility of the study of the Temperaments.

" It contributes to the establishment of the foundations of clinical medicine. Temperaments are seldom mentioned in books, but much cared for in practice, for they must be considered in the diagnosis and treatment of every disease. Organic tendencies are in fact the primary conditions of diseases : they determine their nature and form, regulate their course, and augment or limit their intensity. It is upon these tendencies that the physician bases his estimate of probabilities of the progress and issue of the disease, and the effects and doses of appropriate medicine. All predisposing causes, *i. e.* those which it is most essential to be apprized of, originate in the different temperaments of the economy. How are we to oppose the development of these causes, if the very principle of the temperament is unknown to us ? The important point is to arrive at the *idiosyncracy* by a practical and searching knowledge of the temperament. The practitioner who joins to the experience of things that of persons, possesses a safe basis for appreciating the unequal proportions of vital energy we remark among most men. The sagacity of judgment, the certainty of tact, the almost infallible experience of some practitioners, oftentimes only depend upon their precise knowledge of idiosyncracies and temperaments."

We regret that want of space will prevent our offering any analysis of the author's papers on *Moral Medicine*, and the *Basis of the Progress of Medical Science*. The former, however, was noticed by us on its first publication (M.-C. Rev., No. 70). We will occupy the remainder of this notice with a few passages selected from the " *Galerie Medicale*," which contains some masterly sketches of the characteristics of several celebrated members of the profession, as Alibert, Desgenettes, Broussais, Marc, Richerand, Larrey, Corvisart, Boyer, Dupuytren, &c. &c. We should feel much pleasure if it were in our power to re-produce these *in extenso*, for they seem to have been very conscientiously written. " This gallery," says the author, " I make bold to say, is a true study of man, for in the lives of some celebrated practitioners of our epoch, I have sought less for details of interest than for lessons of profit and examples for instruction." The absence of a collection of biographies has always seemed to us a great and leading defect in our medical literature. Many of our practitioners know little or nothing of the lives and doctrines of their predecessors, and are unable to take encouragement or warning, as the case may be, by their example. A volume of well-written medical biographies would, we doubt not, prove an attractive as well as an useful work. Nothing worthy of the name has been attempted in this, or indeed in any country.

*Dupuytren.*

The determined and overbearing character of this celebrated surgeon manifested itself from the very commencement of his career. When, at

17 years of age, he was appointed one of the Demonstrators at *l'Ecole de Medicine*, his poverty was so great that he was obliged to mend his own clothes. So far from despairing, however, he had such a complete conviction of his own superiority, and presentiment of its successful exertion, that he had already in view the surgical dictatorship which he afterwards attained. The renown of Bichat was a source of continual vexation to him, and upon hearing of his death he exclaimed, "I begin to breathe." From this period, too, his reputation began to increase rapidly.

"Knowledge, judgment, indefatigable industry, untiring patience, address, energy, and resolution, were all employed by him in the manner characteristic of a man who is determined to succeed—one who is never at ease but at the head of the crowd, who proceeds straight to the end in view, in spite of all the obstacles and difficulties of rivalry. This is in fact the period at which Dupuytren really worked, when he amassed most of his scientific labours, although it was long before he reached his eventual eminence. There was then to be observed in him the decided desire of increasing the riches of science, of extending its limits, united with that burning ambition which destroys rest, and which is the foundation of that strength which characterizes the man impatient of all controul and jealous of every influence."

His avowed determination in 1812 to obtain the professorship held by Sabatier in *l'Ecole de Medicine* raised him numerous enemies and opponents; but he triumphed over all, in spite of an evident desire of the judges to favour some of these. Nothing now impeded his progress.

"Arrived at the apogee of his renown, the genius of Dupuytren was quite equal to his position; and he was enabled to struggle with the torrent of occupations by which he was surrounded. There was indeed something really gigantic in that intelligence which provided for everything by its prodigious activity, indefatigable industry, determined ardour, and by a sort of ubiquity which allowed him to accomplish as well as to desire everything. For twenty years he was always one of the earliest at the *Hôtel-Dieu*, however inclement the season. He passed more than five hours there in the severest bodily and mental exertion. No sooner had he left these than new duties and crowds of patients called him to all parts. * * * * * * * * Whoever wishes to give unrestricted praise to Dupuytren must consider him in his professor's chair. There he triumphed—there he excelled. It was a remarkable thing to hear this illustrious surgeon delivering his beautiful lectures on Clinical Surgery, amidst three or four hundred silent and attentive pupils. With what order and method he enchained the attention of his auditors, how accomplished was he in instructing them, exhibiting the light of evidence with a skilful and vigorous logic, and inculcating good and sound information. His clear, calm, strong delivery, lucid and expressive, fluent, easy without prolixity, elegant without effort, always fortified by things and facts; the clearness of his views; his rare talent for exposition and deduction; his astonishing facility in defining, classifying, and expressing his ideas, in rendering objects sensible and evident, and in arresting, fixing, and directing the attention, gave to his lectures a singular attraction. No idle detail, declamation, or ornament was there: all was clear, precise, intelligible, all tended to fact, to its object, and to instruction. * * * * * * Nevertheless, the pupils sometimes reproached the professor with dwelling only with predilection upon those cases in which success had crowned his efforts, and disguising his reverses with skill. Another, and perhaps better-founded, reproach is, that this illustrious professor never quoted any one else, and French surgeons still less than any. To speak the truth, he had a marked and exclusive disdain for all that had not proceeded from himself; and this indifference, or rather scepticism, for every thing and every body, when the talent of his rivals was in question, had its root in his naturally haughty character—completely despising the opinions of others—those of the past as useless, and those of the present as inferior.

"What I have said of the professor exhibits his qualities as a surgeon. It is allowed by all that Dupuytren was the most remarkable man in the profession of his epoch. Few have possessed that vast and rare assemblage of qualities which constitute a great surgeon. He had especially an admirable strength of judgment, which rendered his diagnosis so true and affirmatory, that it struck every one with astonishment. Moreover, in the observation and application of precepts, there was an exactness of analysis, a certainty of practical good sense, and a depth of examination, which are certainly rare. Although bold, without the least trace of pusillanimity, he never operated until the last moment, when he was certain that this furnished the last recourse of art. * * * * * * * Arrived at the height of his reputation, Dupuytren desired to be not the chief but the tyrant of French surgery; and the most exclusive and bitter individuality dominated his entire being, dictated his thoughts, and governed his actions. So, in his social relations, he much preferred unexacting friendships and passive unconditional devotion.

"Justice obliges us to add, that the high faculties of this great surgeon were not those which may be termed transmissible. He had not a genius for discovery but for application. This leaves but little harvest for succeeding generations to reap. From such great talent and renown one would expect an imposing succession of works which would add to the acquirements of science, and long leave their traces there. Alas! what remains to us of Dupuytren, what his biographers will carefully collect, is not to be compared with that which we had a right to hope for. It is this which has given rise to the saying that, after all, this great man was only a famous man. * * * * * * * In studying this celebrated surgeon with care, we may, to a certain extent, explain this defect of accordance between his labours and talent, and the insignificant amount of results. He possessed much more of the talent for teaching, and all that required the aid of speech and action, than for slow, continuous, labour. Next, he had embraced the whole circle of surgery. He was not one of those who devote their lives to the production and realization of an idea. He wished to know and distinguish himself in every part of his profession, whence it resulted that he only contributed here and there to the progress of some portions of surgery. Add to this that, desirous of becoming the first in all, he wished to accomplish this by the aid of a large fortune, and the amassing this occupied him incessantly, especially as this was a natural bent of his disposition."

A lofty pride seemed to have been his distinguishing feature, which accompanied him in every act and word, and the inordinate ambition hence arising doubtless was one of the causes of his merit and renown. To serve this ambition, however, he would condescend to almost any meanness and duplicity, so as to impart to his character, observed for any length of time, the most strange contrasts. When his object was to be answered by it, it was painful to see "the insinuating air, the wheedling suppleness of so proud and irritable a man." He was surrounded by flatterers, but he was also exceedingly susceptible to the attacks and criticisms his own disdainful conduct provoked.

"Criticism irritated him as an injury, while blame tormented him as an intolerable novelty. Admirers and acquaintances he had, but few or no friends, and he found the surgical crown, like others, was not without its thorns. His genius, talent, and dignities were envied, but no one was ignorant that beneath this brilliant surface were anguish and chagrin. He was looked upon as a sort of power whom one must prevent from becoming an enemy, but with the impossibility of ever becoming his friend. Every one stretched a hand to him, but no one shook his cordially. He was at once admired, feared, and blamed—a singular destiny for a man of such incontestable superiority. Let us add that, like other proud, susceptible, and irritable beings, he was unequal and changeable. Literally, to use the vulgar expression, one did not know which end to lay hold of him by. He did good and evil by starts and caprice. If, in some cases, he might be taxed with falsehood and harshness, in others, he exhibited an exemplary loyalty and unheard-of disinterestedness. Although he felt little disposed to oblige those who surrounded him, we find him one day rising at four in the morning to go to Bicetre to solicit the favour of Murat for a friend. This strangeness of character equally influenced his general bearing and even personal appearance. Who does not recollect his strange, old-fashioned gaiters, his everlasting, worn-out green coat, his seldom-shaved beard, his apron up to his arm-pits, and the roll which he munched while driving from place to place in a shabby hackney-cab?"

With all this affectation of simplicity he coveted scientific distinctions, and was especially fond of his title of *Baron*. Probably from the obstacles to his progress which he found his poverty to offer in early life, he acquired an insatiable love of money, and became immensely rich. Having heard that Astley Cooper possessed six millions of francs he would never rest until he had accumulated the like. To this end he sacrificed all the time that might have been devoted to the advancement of science; and even his Clinical Lectures would not have been preserved but for the care of some of the auditors. Dupuytren himself published nothing, except a wretched letter in the newspapers of 1832, announcing decoction of poppy-heads a cure for the cholera! His riches, however, brought no satisfaction, but only the desire to add to them; he was not organised for happiness and never possessed it. He died aged 58. His will, M. Parise says, does high honour to him, but he does not state its dispositions.

### *Broussais.*

Entering life amidst the turbulence of the French Revolution, the early part of the career of Broussais was stormy and uncertain. Soldier, corsair, hospital-clerk, navy-surgeon, student, civil practitioner, and lastly, military surgeon in the Napoleon campaigns, his existence was destitute of repose. Nevertheless, robust in body, firm in determination, and gifted with great aptitude for work, he pursued his studies in spite of every fatigue and difficulty. He eventually came to Paris and became a pupil and an intimate of Bichat, and an ardent admirer of the opinions of Pinel. His treatise *Des Phlegmasies Chroniques,* which had cost him immense labour, fell almost still-born from the press; but, although he felt the disappointment in the keenest manner, he concealed it, and pursued his indefatigable researches in Spain and Italy, examining thousands of bodies, accumulating a vast mass of facts, and meditating over them for years.

"It was in 1815 that he commenced his private course. It was only two paces from the Faculty that he cast forth his haughty defiance and raised altar against altar, doctrine against doctrine. From the first he announced himself as a reformer of science, proceeding by physiological demonstration to a complete rebuilding up of the structure of medicine. And let it not be supposed his course resembled any other of this kind, that is a simple and calm exposition, methodically elaborated, of the principles of the art. No, it was literally a true arena where the professor fought alone and to the death. It was on his part an exhibition of harshness and frankness, an aggressive and ill-boding discussion of received theories, a continued explosion of recrimination against the science of formerly and its constructors, a constant affirmation of his own doctrines, a haughty and disdainful treatment of those of others—the like of which had never yet been seen. Then, the choleric argument, the passionate tone, abrupt gestures, the thundering voice of the professor, and his animated countenance, for he always seemed as if illumined with inspiration, added much to the effect of his addresses. The numerous and crowded auditory kept the deepest silence, the more as their attention was always, so to say, enslaved, because it was continually excited. * * * * Broussais joined to a great penetration a very cultivated mind; and thus we may remark in his lectures, in his reiterated appeals to the progress of science, a skilful mixture of truth and error, a certain art of presenting the latter with the probabilities of reality. Although exclusive in his ideas he announced solid principles. Not content with observing and collecting many facts, he knew how to compare them, and deduce from them consequences often strained, but at other times undoubtedly just. To consider science under aspects until then unknown; to open a new horizon to ulterior researches in the study of diseased organs; to state his principles in an exact and precise manner; to reduce them to the rigour of a theorem; such was the end he proclaimed, and which he flattered himself he attained. So much knowledge and application, such high pretensions, united to the fire of conviction which seemed to animate him, to that ardour of proselytism, to that perseverance in self-glorification, to that fanatical esteem of his system, perpetually announced as the complete *resumé* of the entire range of medical truths, gave to the lectures of Broussais an astonishing popularity. Auditors crowded and pushed their way to them as to a theatrical amusement, and, more than once, pupils, who were unable to gain admission, might be seen taking their notes in the sun or rain when the loud tones of the professor allowed them to seize the purport of some of his observations."

In 1815 his *Examen* appeared. In this, the opinions of his adversaries were handled with the bitterest sarcasm and irony, and his own doctrine developed with surpassing skill. His success was prodigious. Not only were his dogmas received with enthusiasm by the schools, but even the convictions of elder practitioners were much shaken; so that the advent of a new era of medicine was proclaimed on every side. The deceptive simplicity of the system, and the magical words "progress," "advancement of science," "new doctrine," contributed immensely to its popularity; and those who dared attempt to oppose its reception were covered with terms of reproach and contempt. It is not surprising that Broussais became intoxicated with the incense constantly poured out before him, and imagined himself exalted into supreme judge of what should or not be received as sound doctrine. Yet, at the end of a few years, it was discovered that he, like many other reformers, was more potent in criticizing and destroying present structures than in raising new ones; and that the system of the man who had been so ready of visiting those of others with sarcasm and contempt, was not itself proof against the shafts of the critic. Remarks, criticisms, exceptions, and objections now multiplied on every side; but we need not follow the exposure of the errors of a system which never obtained much currency here, notwithstanding the Gallo-mania which some of our Journals endeavoured to foster. M. Parise exhibits concisely not only the unscientific character of the principles laid down, but the bad effects which too frequently flowed from their practical adoption; for a practice which had been borne by the robust young soldiers coming within the sphere of Broussais' observation, was ill-suited to the feebler constitutions so often met with in civil practice. Once submitted to a rational examination, the system rapidly declined in estimation; and even when in the possession of an official professorship its constructor could not raise it from the neglect and disfavour into which it had fallen. His vehement denunciations against the older systems no longer drew the crowded auditories of heretofore, and the few pupils who attended his lectures seemed to do so more as a curiosity of bye-gone days than as a reality of the present. Whether from faith in its truth, or from the desire of drawing upon himself the public attention which had so notoriously flagged, Broussais next became a propagator of phrenology. Again he had a large and applauding, if not a judicious auditory: "but the concessions he made to his adversaries were so numerous, that Gall would hardly have recognized his own doctrine in his discourses." He adopted for the defence of this the same paradoxical arguments, the mixture of error and truth by which he had sought to defend the doctrine of irritation. It will not seem surprising that Broussais only attained a rank in the practice of the profession very disproportionate to his merits and reputation. His exclusive mode of viewing cases so limited his range of therapeutical indications, although the force of circumstances sometimes obliged him to make concessions. One of those related of him is amusing enough. A patient having declared to him that he could support his regimen no longer, and that he was literally dying of hunger, Broussais reflected for a moment and replied, "very well, you carnivorous animal, I will satisfy you," and ordered him a spoonful of broth in a glass of water. However, concessions of any kind were wrung from him with difficulty, for he prided himself upon his inflexibility to objections or adversaries. Nevertheless, after venting the most bitter sarcasms on places and riches, he allowed himself to accept some of these, and the great agitator was found lecturing in the professor's gown, and placing six lines of honourable titles after his name in one of his works!

Regarding Broussais with impartiality, uninfluenced by the excessive praise of his admirers or the bitterness of his detractors,

"We shall find he was a man of rare capacity and incontestable merit. It will be allowed that he has rendered services to science and has proclaimed truths which will endure. Nevertheless, if he appears great, the narrowness of his views is striking when compared with those of Bichat. This is because he was too exclusive, too absolute, in his ideas, because he has forcibly referred facts to two or three principles only, that is to either too few or too many, and because he has, with a view to his system, proclaimed the true and the false with an equal ardour. In the place of pruning, adding, and extending, he was desirous of radically destroying and re-constructing; and, at a period when doubts were raised, he struggled for the most formal materialism. Thus, although he has shed a luminous ray on science, although he proclaimed himself its master, his opinions have been attacked, his pedestal shaken, and his titles to true glory strongly contested. What remains of him now? Has Broussais done what he ought and could? Has he fulfilled his mission worthily and completely? Posterity, that high court of appeal for reputations, will decide in time to come. She will be just, yet severe; for, according to Scripture, much is required from him to whom much is given."

*Chervin.*

Nicholas Chervin is one of the men of science of whom France has just cause to be proud, and we regret our limits forbid our availing ourselves at any length of the glowing picture of his devotedness furnished by M. Parise.

He was intended for commercial pursuits, but happening to accompany a friend at Lyon to an anatomical lecture, he was so delighted with the lucid description furnished of the difficult bone, the *sphenoid*, that he at once resolved to devote himself to the study of medicine. He entered the army, but at the Peace came to Paris. At a breakfast party, consisting of medical men, *the contagiousness of the Yellow Fever* became the object of debate, and Chervin observed that, as far as he had read on the subject, he disbelieved in its contagion. "What can you know about it?" replied one of the company. "I can furnish some proofs in support of my opinion," said Chervin. "Truly," added his adversary, "these will never be obtained on the banks of the Seine." Chervin felt himself hurt by this sally, and to such an extent that, from that moment his money, time, labour, and entire life were devoted to the elucidation of this question. At a period when the Yellow Fever was an object of dread amongst medical men, he at once sailed for America to visit the enemy in its sources and fastnesses, and the narration of his labours, exertions, and dangers, would occupy pages in their recital. Wherever the disease had prevailed or was prevailing he bent his steps, no matter at what cost of time or risk of health, and took up his temporary residence in even the most pestilential districts. Frequently he tasted the black vomit, and he arduously pursued anatomical investigations under a tropical sun, with a temperature of 106°. Nothing alarmed, nothing arrested him. He accumulated an immense mass of authentic documents properly testified, and returned to Europe. The yellow fever, however, then breaking out in Spain, he at once repaired thither, and thence returned to Paris, bearing with him his documents, and the unalterable conclusion, to the practical carrying out of which he devoted the rest of his life, "that the yellow fever is of paludial origin, its violence and gravity resulting from severity of climate; that it is not contagious; and that lazarets and quarantines should be considered onerous to governments, and especially injurious to commerce."

His desire was to bring his documents enforcing this doctrine before the public, and to enable him to do this he was content to submit to any privation. He took a wretched apartment in Paris for *six months*, and inhabited it for nearly *nineteen years*. He knew the interests and prejudices he had to contend with, but never despaired of ultimate victory. He was always prepared for every adversary, and overwhelmed him with documents, proofs, and authorities, whose refutation was not a little difficult. The truth before everything was his maxim, and he refused to bend to any authority in opposition to this. A man of this stamp, active in the pursuit of what he considered a duty to humanity and to the well-being of his country, and who advanced his arguments with the bold tones of one convinced of their unalterableness, of course met with numberless opponents. He was denounced as a visionary, an innovator, almost as an anarchist. "I am strong enough," he proudly exclaimed, "to brave injustice, ingratitude, ill-nature, and even poverty, that implacable enemy to social man. I can support all this." This was no vain boast. He spread his essays, reports, letters, memoirs, &c. on every side; and eventually he was compensated by the Academy, after a long and vehement discussion, affirming his propositions. A few years after, the Monthyon Prize was decreed him, and in 1832 he was chosen a member of the Academy by a large majority, the editors of several journals also freely opening their pages for the propagation of his opinions. He had the pleasure, also, of finding the Government modifying their quarantine system in 1835 and 1839, and the still greater one, of finding opinion setting in stronger and stronger against the system altogether. But he was not the man to be satisfied with half success, and continued his efforts as perseveringly as ever to the last. He lived to witness no further results of his labours, nor even to present his various documents and publications in any luminous body of doctrine, his time being, indeed, chiefly occupied in its active polemical defence.

"Chervin was, indeed, one of those models of severe morals of ancient times. His moral organization was quite different from that of the ordinary type. We may say he was a honest man in all the greatness and magnificence of the expression; for he possessed that golden flower of good faith, rectitude, and judgment which forms its base and root. When he was seen to labour, demand, solicit, even intrigue, to spare neither distance, trouble, writing, or expense, who would not have believed he was acting for himself and his own affairs, in order to obtain some reward or lucrative employment? Nothing of the kind: the object of so much care was the public good. He was concerned with the advancement of science, the enlightenment of authority, the economising the finances of the State, and the saving of millions to commerce, while he himself was in want of everything! So will his life always be a great example for men who fully devote themselves to the good of the human race, and the culture of science. There will be found an example of self-abandonment, of devotion the more striking as it is original, and so to say *sui generis.* * * * * * * Having neither patrimony, place, practice, or powerful protectors, simple in his tastes, content with little, uniform in his life, he had no other affection, no other passion than that for the yellow-fever, which he had, as it were personified, and which was called his companion or *heroine.* All else was equal or indifferent to him. Thus he wanted for everything, for the small pension from the Academy was his only resource. During his latter years, he had to struggle with the three greatest afflictions of humanity—disease, old age, and poverty, triple Euminides, against whom men are usually devoid of energy. But Chervin was always the same. Adversity found him armed with strength and patience; there was nothing changed in his views, his habits, or his labours. No complaint or recrimination escaped him, whether in depreciation of what is, the elevation of what ought to be, or against men the most favoured by fortune. Never did he manifest jealousy or envy of his brethren, or cast a longing eye upon an opulent position. Never was he exposed to the torture of that *medical bile,* so acrid and so corrosive in some cases. Excepting as regards the scientific point of the *non-contagion of the yellow fever,* he knew how to suffer and be silent."

He resided in a miserable garret without even the necessaries of life; but who among us does not envy his honorable poverty, with his table and few chairs, his books arranged upon two planks, and his *enormous dusty trunk,* containing his precious documents, "collected at the price of his fortune, his rest, his health, and his life in every part of the globe," rather than the prosperous celebrity of some of his contemporaries? He was attacked with hemiplegia in 1842, and died the following year.

*Larrey.*

The character and services of Larrey are so well known and appreciated in this country, that we need borrow little from the sketch here drawn by his friend and colleague. It is always, however, pleasing to accumulate instances of the excellent conduct of the members of our profession; and we question if the *beau-ideal* of a military surgeon was ever more realized than in the character of this celebrated man.

"At the battle of Eylau he remained 30 hours without eating; and so hurried and ardent was he in dressing wounds and giving orders, that paralysis of the bladder was nearly induced. Friends, enemies, every one, might claim his services. He saw only suffering creatures imploring the succour of art. Degree of rank in nowise decided his preferences. No matter what the epaulette, he only saw the wounded man, whose blood, poured out for his country, was equally noble and precious. After having operated on Marshal Lannes, and visited Duroc, the intimate friend of Napoleon, he would dress with equal eagerness the wounds of the lowest soldier or of the conscript of yesterday. His colleagues even, always his dearest friends, never met with preference. Our honorable *confrere*, M. Tanchou, tells us that, when wounded in the battle of Montmirail, he was taken to Larrey's *ambulance*: 'Your wound is a slight one,' he said, 'we have only room and straw here for bad accidents. However, they may put you in yonder stable.' The gravity of a wound was in fact the only ground of preference in his eyes. * * * * * * * * * * The more severe and doubtful an action became, the more the activity and resources of this great surgeon multiplied—his first and only thought being the succour of the wounded. During the highest raging of the battle of Waterloo, when the fire was continuous and murderous, the author of this sketch and Larrey met, when he exclaimed, 'My dear colleague look to your wounded soldiers. Pay attention only to them.' He added, 'the firing is hot indeed, but let every one do his duty and all will go on well.' * * * * * * * Larrey had so little dissimulation, that one of his defects, perhaps the only one, was the high opinion he entertained of his own talent and works. He praised himself with such a good faith and confidence, but his weakness was the more excusable, as it was not without foundation, facts answering to words. Frequently a deep colossal pride, peculiar to celebrated men, is disguised without, and hidden with care; but Larrey concealed nothing. All his thoughts and words were without disguise, for he knew not how to conceal his pride at the bottom of his heart, like some who are termed modest. Oftentimes he raised a pedestal to himself with the same simplicity, the same confidence, as if he had performed the most virtuous action or the greatest sacrifice. We may sometimes smile at his simple and frank want of modesty, and at the same time admire his noble heart full of rectitude, honour, probity, and courage, a stranger to the manœuvres of personal interest, concealed under the veils of philanthropy and love of science. What he delighted in, above all things, was to protect and assist others by his counsels and his credit; so that he always regarded surgeons whom he had known, and who were a little younger than himself, as his pupils. In writing to the late M. Coutanceau, then professor at *Val de Grace*, Member of the Academy, and author of different works, he never omitted concluding with 'your affectionate brother-in-law and former professor.' This was not pride or the desire of superiority, but a bond of patronage, goodwill, and reciprocal attachment, which he delighted in tightening and continuing."

In perusing the accounts of the lives of late and present members of our profession in France and in our own country, we have often been struck with the far larger proportion in the former nation who have raised themselves from indigence and comparatively low conditions to the highest position of professional eminence. And in the facilities which that country affords to her industrious and enterprising youth, destitute of family connections, pecuniary resources, or hospital influences, for attaining the highest offices in her power to bestow, we see an example well worthy of imitation in our own country, where a very different system of selection, or rather of succession, prevails.

